# Immunofluorescence microscopy of SNAP23 in human skeletal muscle reveals colocalization with plasma membrane, lipid droplets, and mitochondria

**DOI:** 10.14814/phy2.12662

**Published:** 2016-01-05

**Authors:** Juliette A. Strauss, Christopher S. Shaw, Helen Bradley, Oliver J. Wilson, Thierry Dorval, James Pilling, Anton J. M. Wagenmakers

**Affiliations:** ^1^ Research Institute for Sport and Exercise SciencesLiverpool John Moores UniversityLiverpoolUK; ^2^ Centre for Physical Activity and Nutrition ResearchSchool of Exercise and Nutrition SciencesDeakin UniversityGeelongVictoriaAustralia; ^3^ School of Sport, Exercise and Rehabilitation SciencesUniversity of BirminghamEdgbastonUK; ^4^ Institute for Sport, Physical Activity and LeisureCarnegie FacultyLeeds Beckett UniversityLeedsUK; ^5^ Discovery SciencesAstraZeneca R&DCambridgeUK

**Keywords:** Glucose transporter 4, intramuscular triglyceride, lipid droplets, mitochondria, synaptosomal‐associated protein 23

## Abstract

Synaptosomal‐associated protein 23 (SNAP23) is a SNARE protein expressed abundantly in human skeletal muscle. Its established role is to mediate insulin‐stimulated docking and fusion of glucose transporter 4 (GLUT4) with the plasma membrane. Recent in vitro research has proposed that SNAP23 may also play a role in the fusion of growing lipid droplets (LDs) and the channeling of LD‐derived fatty acids (FAs) into neighboring mitochondria for *β*‐oxidation. This study investigates the subcellular distribution of SNAP23 in human skeletal muscle using immunofluorescence microscopy to confirm that SNAP23 localization supports the three proposed metabolic roles. Percutaneous biopsies were obtained from the *m. vastus lateralis* of six lean, healthy males in the rested, overnight fasted state. Cryosections were stained with antibodies targeting SNAP23, the mitochondrial marker cytochrome c oxidase and the plasma membrane marker dystrophin, whereas intramuscular LDs were stained using the neutral lipid dye oil red O. SNAP23 displayed areas of intense punctate staining in the intracellular regions of all muscle fibers and continuous intense staining in peripheral regions of the cell. Quantitation of confocal microscopy images showed colocalization of SNAP23 with the plasma membrane marker dystrophin (Pearson's correlation coefficient *r* = 0.50 ± 0.01). The intense punctate intracellular staining colocalized primarily with the mitochondrial marker cytochrome C oxidase (*r* = 0.50 ± 0.012) and to a lesser extent with LDs (*r* = 0.21 ± 0.01) visualized with oil red O. We conclude that the observed subcellular distribution of SNAP23 in human skeletal muscle supports the three aforementioned metabolic roles.

## Introduction

Defects in insulin‐mediated glucose uptake in skeletal muscle contribute to whole‐body insulin resistance in obesity and precedes the development of type 2 diabetes. The mechanisms resulting in obesity‐induced skeletal muscle insulin resistance are only partially understood. A paradox that has provoked much research in muscle physiology is that intramuscular triglycerides (IMTG) accumulate and are positively associated with insulin resistance in obese, sedentary individuals, but are even higher in endurance‐trained athletes who are highly insulin sensitive. This discrepancy is called the athlete's paradox (Goodpaster et al. 2001; van Loon 2004). The current consensus is that in endurance‐trained athletes a high capacity for fat oxidation and IMTG resynthesis in the period after exercise prevents the accumulation of fatty acid (FA) metabolites, such as long‐chain acylCoAs, diacylglycerols, and ceramides, which have been proposed to induce insulin resistance and reduce skeletal muscle glucose uptake (Ellis et al. 2000; Itani et al. 2002; Kim et al. 2004; Amati et al. 2011; Dube et al. 2011). Synaptosomal‐associated protein 23 (SNAP23) is an understudied protein in skeletal muscle that may link excessive storage of lipid droplets (LDs) in obese and sedentary individuals to impairments in insulin‐mediated glucose uptake via multiple mechanisms.

SNAP23 is one of the 36 SNARE (Soluble N Ethylmaleimide Sensitive Factor Attachment Protein Receptor) proteins (Jahn and Scheller 2006). Each of which share a characteristic SNARE “motif” in the form of a conserved sequence of 60–70 amino acids. The SNARE motifs mediate SNARE complex formation, which are important in many intracellular docking and fusion processes. SNAREs are categorized according to the role they play in membrane fusion; vesicle‐SNAREs (v‐SNAREs) are found on the transport vesicles and target‐SNAREs (t‐SNAREs) are found at the plasma membrane. SNAP23 is a t‐SNARE (for a review see Jahn and Scheller 2006), 210 amino acids in length, which is expressed in skeletal muscle. In its role as a SNARE protein, SNAP23 is able to bind with v‐SNAREs, VAMP1 and 2 and also syntaxin1, 2, 3, and 4. Together these SNARE proteins play key roles in the mechanisms that lead to targeted exocytosis. In line with its role as a t‐SNARE, SNAP23 along with these other SNAREs, has been shown to mediate insulin‐mediated GLUT4 docking and fusion with the plasma membrane (Foster et al. 1999; Kawanishi et al. 2000).

Lipid droplets in skeletal muscle provide a storage depot for excess lipids and a readily available fuel source during exercise in trained humans (van Loon 2004; Shaw et al. 2010). Recent evidence obtained in vitro has suggested that SNAP23 may also play divergent roles in intramuscular lipid storage and the channeling of LD‐derived FA into mitochondria for *β*‐oxidation. Both processes can influence insulin action as they keep the concentration of FA metabolites low. During lipid oversupply, as occurs in sedentary obese individuals, it has been proposed that SNAP23 is sequestered away from the plasma membrane to the intracellular LDs where it aids their growth through LD fusion (Bostrom et al. 2007). These findings have led to the hypothesis that in lipid‐induced insulin resistance, LDs would hijack SNAP23 from the plasma membrane and prevent fusion and docking of GLUT4 storage vesicles thus leading to impaired insulin‐stimulated glucose uptake in skeletal muscle and impaired glucose tolerance (Sollner 2007; Bostrom et al. 2010).

Transmission electron microscopy and confocal immunofluorescence microscopy have both shown that LDs are localized in close proximity to the mitochondrial network in skeletal muscle (Hoppeler 1999; Shaw et al. 2008). Electron microscopy has also shown that a 7‐week endurance training protocol in previously untrained individuals (Tarnopolsky et al. 2007) led to an increased spatial contact between mitochondria and LDs. This increased spatial contact in trained individuals has been suggested to aid in the efficient oxidation of FAs liberated from lipid droplets upon lipolysis during exercise as it reduces the diffusion distance of the released FAs to the site of *β*‐oxidation. In a more recent study in fibroblasts, ablation of SNAP23 resulted in a decreased spatial complex formation between mitochondria and LDs and decreased mitochondrial *β*‐oxidation (Jagerstrom et al. 2009). This has led to the suggestion that SNAP23 is implicated in the formation of functional LD‐mitochondria complexes, specifically helping to shuttle LD‐derived FAs into the mitochondria for *β*‐oxidation (Jagerstrom et al. 2009).

Given the proposed roles of SNAP23 in muscle glucose uptake, LD fusion, and channeling of FA released by LD into mitochondria for *β*‐oxidation, this protein seems to play an important role in the mechanisms that lead to impaired glucose uptake, impaired intramuscular lipid metabolism, and insulin resistance/type 2 diabetes (Sollner 2007; Bostrom et al. 2010). The purpose of this study was to use an extensively validated antibody targeting SNAP23 to describe for the first time the fiber‐type distribution and subcellular localization of SNAP23 in human skeletal muscle, in particular in the context of the three proposed roles of SNAP23 described above. The developed staining methodologies are important as they generate the first detailed confocal images of the distribution of SNAP23 in human skeletal muscle. The study was conducted using percutaneous muscle biopsies obtained at rest, in the overnight fasted state from six lean, healthy males and sections were analyzed using newly developed and validated widefield and confocal fluorescence microscopy methods.

## Materials and Methods

### Subjects and pre‐experimental procedure

Six lean, moderately active males were recruited to this study (age [years]: 20.2 ± 0.9, BMI [kg/m^2^]: 22.4 ± 0.4). Subject characteristics are listed in Table 1. Approval for execution of the studies was obtained from the West Midlands Solihull NHS Research Ethics Committee. Written informed consent was obtained from all participants. Prior to the experimental trials, subjects completed a progressive exercise test to exhaustion in order to determine their maximal oxygen uptake (VO_2max_). Subjects cycled on an electronically braked cycle ergometer (Lode BV, Groningen, The Netherlands) in order to determine maximal oxygen consumption using an online gas collection system (Oxycon Pro, Jaeger, Wuerzburg, Germany). The test consisted of initially cycling at 95 W, followed by sequential increments of 35 W every 3 min until exhaustion. VO_2max_ was taken as the highest value obtained in the last 30 s of the test.

**Table 1:** Subject characteristics

	Lean male participants
*n*	6
Age (years)	20 ± 1
Height (m)	1.79 ± 0.03
Body mass (kg)	72.03 ± 2.75
Body mass index (kg/m^2^)	22.4 ± 0.4
VO_2_ max (mL/min/kg)	55.8 ± 1.8
Wmax (W)	282 ± 17

Data are presented as means ± SEM.

### Muscle samples

#### Sample collection

Percutaneous muscle biopsies were obtained from the *m. vastus lateralis* under local anesthesia (~5 mL 1% lidocaine) using the percutaneous needle biopsy technique (Bergstrom, 1975). Following local anesthesia, a small (~1 cm) incision was made in the skin and the fascia covering the central portion of the *m. vastus lateralis*. The biopsy needle was then inserted and sequential cuts were made with the application of suction (~100 mg). Following removal, samples were blotted of excess blood and any visible fat and connective tissue was separated from the sample and discarded. Part of the biopsy sample (~30 mg) was embedded in Tissue‐Tek OCT Compound (Sakura Finetek Europe, Zoeterwoude, The Netherlands) on a cork board which was immediately frozen in liquid nitrogen‐cooled isopentane (Sigma‐Aldrich, Dorset, UK) and stored in precooled cryotubes at −80°C for histologic analyses. The remainder of the biopsy samples was snap frozen in liquid nitrogen and stored at −80°C for subsequent Western blotting analyses as part of the antibody validation.

#### Immunofluorescence analysis

Cryosections (5 *μ*m) were cut using a microtome (Bright Instrument Company Limited, Huntingdon, UK) housed within a cryostat at −25°C. The sections were collected onto uncoated, precleaned glass slides (VWR International Ltd, Leicestershire, UK). Two cryosections from two participants were mounted onto the same slide to reduce the variation in staining intensity between the sections. Slides were fixed for one hour in 3.7% formaldehyde. Following 3 × 30 sec rinses in doubly distilled water (dd H_2_O), the slides were permeabilized in 0.5% Triton X‐100 for 5 min. Subsequently, slides were washed 3 × 5 min in phosphate‐buffered saline (PBS, 137 mmol/L sodium chloride, 3 mmol/L potassium chloride, 8 mmol/L disodium hydrogen phosphate, and 3 mmol/L potassium dihydrogen phosphate, pH 7.4), after which the sections were incubated in appropriate primary antibodies for 2 h. Following a further 3 × 5 min wash in PBS, secondary Alexa Fluor fluorescent‐conjugated antibodies were applied for 30 min. When visualizing IMTG, the neutral lipid dye oil red O was used in combination with immunofluorescence using the method of Koopman et al. (2001). Oil red O was applied to the sections for 30 min (following 3 × 5 min washes in PBS). Slides were then rinsed 3 × 30 sec in dd H_2_O followed by a 10 min wash under cold, slow running tap water. Coverslips were mounted to the dried slides using a glycerol and mowiol‐488 solution in 0.2 M Tris buffer (pH 8.5) with the addition of 0.1% 1,4‐diazobicyclo‐[2,2,2]‐octane DABCO antifade medium.

#### Antibodies and dyes

All antibodies were diluted to their predetermined optimal working concentration in PBS with the addition of 5% normal goat serum as a blocking agent. Fiber type was determined using mouse antimyosin heavy chain type I (MHCI) (1:100 dilution, A4.840c, DSHB, University of Iowa, USA, developed by Dr. Blau) followed by the application of Alexa Fluor goat anti‐mouse IgM 594. Fibers stained positively were determined as type I fibers. For staining of IMTG, a working solution of oil red O was made for each staining procedure and consisted of 100 mg oil red O (Sigma‐Aldrich) in 20 mL 60% triethylphosphate (Sigma‐Aldrich). Twelve milliliters of working solution was added to 8 mL dd H_2_O and filtered twice to remove any residual oil red O crystals. The plasma membrane was identified using mouse antidystrophin (1:400 dilution, D8168; Sigma‐Aldrich) followed by goat anti‐mouse IgG_2b_ 594. Dystrophin is a membrane spanning protein complex that is located in the sarcolemma and binds actin to form a connection between the membrane and the cytoskeleton (Haenggi and Fritschy 2006). Mitochondria were stained using mouse anticytochrome c oxidase (COX) (1:50 dilution, 459600; Invitrogen, Paisley, UK) followed by incubation with goat anti‐mouse IgG_2a_ 594. SNAP23 was stained using rabbit anti‐SNAP23 raised against a peptide with amino acid sequence DRIDIANARAKKLIDS, which are amino acids 196–211 in human SNAP23 (1:50 dilution, 111,202; Synaptic Systems, Goettingen, Germany) followed by goat anti‐rabbit IgG 488. Negative controls were included where the primary antibody was omitted (and PBS applied in its place) in order to confirm the absence of nonspecific staining. Furthermore, lack of bleedthrough of signal to alternate channels was determined by viewing the signal of single‐stained sections in opposite channels as described previously (Scriven et al. 2008).

#### Antibody validation

**Figure 1:**
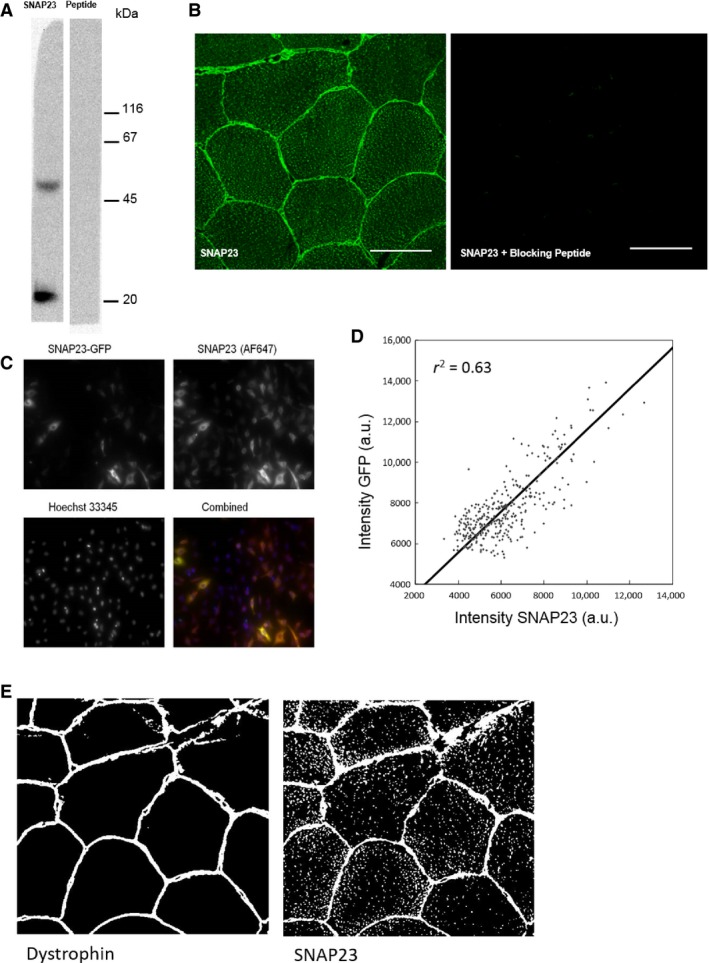
Antibody validation. (A) Immunoblot using anti‐SNAP23 (Synaptic Systems) of a homogenate of human skeletal muscle from a lean male. The molecular weight of SNAP23 is 23 kDa. The band with the higher molecular weight is likely to be a SNARE complex containing at least one molecule of SNAP23. Coincubation of anti‐SNAP23 with blocking peptide eliminated both bands from the blot. (B) Immunostaining of SNAP23 is shown as well as immunostaining using anti‐SNAP23 preincubated with an excess of blocking peptide. Almost all signals are eliminated. Bar = 50 *μ*m. (C) HeLa cells transfected with 100 *μ*g SNAP23‐GFP plasmid incubated for 48 h. The cells displayed a heterogenous expression of the plasmid and show colocalization of SNAP23‐GFP and immunostaining for SNAP23 with anti‐SNAP23 (1:100 dilution). In the merged image the SNAP23‐GFP plasmid is visualized in green, SNAP23 stained with anti‐SNAP23 in red, nuclei in blue, and colocalization of SNAP23‐GFP with anti‐SNAP23 in yellow. Bar = 100 *μ*m. (D) Quantification of the cytoplasmic fluorescence intensities of SNAP23‐GFP and immunostaining with anti‐SNAP23. Data points are obtained from 337 cells in three replicate wells and show a linear relationship (*r* ^2^ = 0.63). (e) Example binary “masks” for the plasma membrane and SNAP23 showing the masks used during the colocalization analysis to identify the “objects” in the field of view (i.e., SNAP23, mitochondria, lipid droplets, or plasma membrane). Bar = 50 *μ*m.

The specificity of the Synaptic Systems SNAP23 antibody was determined using the basic local alignment search tool (http://www.uniprot.org), which confirmed that the target sequence of the antibody was specific in human skeletal muscle for SNAP23 only. Further immunoblot analysis revealed a band at ~23 kDa, the correct molecular weight for SNAP23 (Fig. 1A). The additional band on this immunoblot likely corresponds to SNARE complexes. SNAP23 forms complexes with both itself and other SNARE proteins. These complexes are known to be SDS resistant (Flaumenhaft et al. 1999; Yang et al. 1999) hence an additional band is visible in the immunoblot at approximately twice the expected molecular weight of a single SNAP23 molecule. In a competition experiment in which the SNAP23 antibody was preincubated with a saturating concentration of a peptide against the target sequence of the SNAP23 antibody, all signal was eliminated. This was true for both Western blotting (Fig. 1A) and immunofluorescence methods (Fig. 1B). This supports the suggestion that the additional bands seen on the Western blot are likely complexes of the SNAP23 protein.

The Synaptic Systems SNAP23 antibody was further validated for immunofluorescence applications in green fluorescent protein (GFP) transfection studies. HeLa cells were transfected with human GFP tagged SNAP23 cDNA (Amsbio, Abingdon UK). Cells were incubated with 100 *μ*g SNAP23‐GFP construct for 48 h before fixing in 4% paraformaldehyde for 20 min before washing three times, for 5 min each in PBS. The plate was then washed using PBS and 0.2% triton ×−100. The cells were then stained using anti‐SNAP23 (1 h incubation at room temperature). The primary antibody was applied in the dilution series 1:100, 1:200, 1:500 as well as a control with no primary antibody. This allowed for four repeats on the 96‐well plate. The secondary antibody (donkey anti‐rabbit IgG 647) (Invitrogen) was applied for 30 min at room temperature. The cells were also counterstained with the nuclear stain Hoechst. The plate was then imaged using ImageXpress system (Sunnyvale, CA, USA). The HeLa cells displayed a heterogenous expression of the plasmid. The SNAP23 antibody colocalized with the transfected GFP tagged SNAP23 (Fig. 1C). The antibody was also able to detect variations in SNAP23 as quantification of the cytoplasmic intensities of SNAP23‐GFP and of the immunostaining with anti‐SNAP23 showed a linear relationship (Fig. 1D).

**Figure 2:**
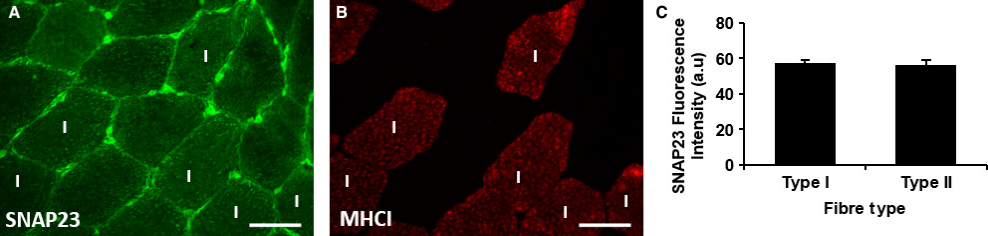
Representative images of SNAP23 distribution in human skeletal type I and nontype I muscle fibers. SNAP23 distribution is shown in cross sections (A) with the slow twitch fiber‐type stain anti‐MHC I (B). Images were obtained using a 40× objective of a widefield microscope. Bar = 50 *μ*m. There was no difference in the fluorescence intensity of SNAP23 between type I and nontype I (C) where type I fibers were positively labeled with anti‐MHCI. Values in panel C are given as means ± SEM.

Before confirming validity of the 111,202 Synaptic Systems antibody, we tested the following commercial SNAP23 antibodies: ab57961 (Abcam, Cambridge, UK), DS‐19 (Sigma‐Aldrich), LS‐C75883 (Lifespan Biosciences, Nottingham, UK). The main criteria that we use for validation of an antibody for use in confocal immunofluorescence staining methods of human skeletal muscle cross sections are as follows: (1) A Western blot using anti‐SNAP23 should show only one single band of the correct molecular weight (23 kD) (if multiple bands are seen they must be justifiable as dimers or SDS‐resistant SNAP23‐protein complexes; see Fig. 1); (2) Confirmation of staining using transfection (e.g. GFP) studies. The antibody should colocalize with the transfected GFP tagged SNAP23; (3) Incubation of the primary antibody with a saturating concentration of the antibody target peptide should eliminate all visible staining; (4) The basic local alignment search tool (BLAST) (http://www.uniprot.org) should demonstrate that the sequence against which the antibody is targeted is only present in the protein of interest in human skeletal muscle. Of the commercial antibodies listed above all but 111,202 (Synaptic Systems) failed to meet at least two of the above mentioned criteria and should, therefore, not be used in immunofluorescence applications to visualize and quantify SNAP23 in human skeletal muscle sections.

#### Fluorescence microscopy

It is important to note that the images in this study were generated using a maximum of two stains. The reason for this is that oil red O has a wide emission spectrum and could therefore not be used to triple stain LDs, mitochondria, and SNAP23, as there would be overlap with the spectrum of the second antibody stain. To measure fluorescence intensity, images of SNAP23 and MHCI were captured using a Nikon E600 microscope coupled to a SPOT RT KE color 3 shot CCD camera (Diagnostic Instruments Inc, Sterling Heights, MI, USA). In order to visualize the Alexa Fluor 350 fluorophores, the DAPI (387/11) excitation filter was used. To visualize the Alexa Fluor 488 fluorophores the FITC (494/20) excitation filter was used. Alexa Fluor 594 fluorophores were observed using the Texas Red filter (595/22). Digital images showing cross sections and longitudinal sections of skeletal muscle fibers were obtained using the 40 × (0.75 NA) objective. The filters used in this microscope system were three excitation filters and 1 dichroic and 1 emission filter (“Pinkel” Triple Set, Semrock, Kettering, UK). The slides were illuminated with a 170 W Xenon Light Source and the filters were controlled using a semiautomated filterwheel (10B 10 Position Filterwheel, Sutter, CA). Detailed digital images demonstrating the cellular distribution of target proteins were obtained using an inverted confocal microscope (Leica DMIRE2; Leica Microsystems, Milton Keynes, UK) with a 63× (1.4 NA) oil immersion objective and used to investigate the colocalization of SNAP23 with dystrophin, oil red O, and COX. The lateral resolution of the HCX PL APO 63×1.4 NA oil objective is 139.4 nm, whereas the axial resolution is 235.8 nm. Alexa Fluor 488 fluorophores were excited with a 488 nm line of the argon laser for excitation and 510–555 nm for emission. Alexa Fluor 594 fluorophores were excited with 594 nm line of the helium‐neon laser for excitation and 601–713 nm for emission.

#### Image analysis

Confocal images to analyze fiber‐type differences in SNAP23 content were processed using Image‐Pro Plus 5.1 software (Media Cybernetics, Rockville, MD, USA). The fluorescence intensity of SNAP23 was measured by selecting the whole of the fiber area and using measures of optical density. Using this method in combination with a fiber‐type stain (anti‐MHCI), it was possible to determine the fiber‐type differences in the SNAP23 signal. All images used for these analyses were oriented as cross sections.

In order to quantify the degree of colocalization between the anti‐SNAP23 staining and the stain used to visualize mitochondria (anti‐COX), lipid droplets (oil red O), and plasma membrane (antidystrophin), we utilized both a Pearson colocalization correlation coefficient (PCC) and a Manders' colocalization analysis. The Manders' colocalization analysis differs from the PCC as it assumes the number of positively stained objects (e.g., SNAP23 and mitochondria) in two fluorescent channels (i.e., green vs. red) is different. For an overview of the two colocalization methods see review by Dunn et al. (2011). The two approaches were used in conjunction as they generate complementary information leading to an improved quantification and understanding of the SNAP23 distribution across the three stained regions (mitochondria, lipid droplets, and plasma membrane). The content of LDs was expressed as the positively stained area fraction relative to the total area of each muscle fiber as previously described in Shepherd et al. (2013). An intensity threshold was uniformly applied to represent a positive signal for IMTG and used to identify LD size.

An average of 14 images was investigated per participant. Each image contained ~7 fibers leading to an approximate total of 98 fibers per participant analyzed for colocalization. The significance of this colocalization was also investigated by overlaying staining of nonmatched sections and analyzing these images using the same method as matched images as described previously by Lachmanovich et al. (2003). For the PCC analysis, the overlap of the two channels (i.e., SNAP23 and the “marker stain” mitochondria, lipid droplets, or plasma membrane) was assessed across the whole image using Image Pro Plus 5.1 software (Media Cybernetics). For the Manders' colocalization analysis (Manders et al. 1993), the two raw images of SNAP23 and the “marker stain” (e.g. SNAP23 and mitochondria) were used to create a “mask” of each channel (i.e., green vs. red) which were then overlayed and the positive signal in one image that overlapped the other image was assessed. An example of a mask of the plasma membrane and corresponding SNAP23 mask can be seen in Figure 1E. The Manders' colocalization analysis was automated using a Matlab^TM^ script and it is of note that no images were rejected from the analysis.

### Western blot analysis

Western blot analysis was performed as part of the antibody validation procedure to confirm the presence of a band at the correct molecular weight for SNAP23. Snap frozen muscle tissue was powdered in liquid nitrogen and transferred to Eppendorf tubes containing homogenization buffer (50 mmol/L Tris‐HCl, 1 mmol/L EDTA, 1 mmol/L EGTA and 1% Triton X‐100 in deionized water). The sample was then homogenized on ice for 30 s using a Polytron homogenizer at slow speed before being mixed with lysis buffer (protease inhibitor cocktail, 10 mmol/L *β*‐glycerophosphate, 50 mmol/L sodium fluoride, and 0.5 mmol/L sodium orthovanadate) and allowed to stand on ice for 2 h. “Debris” containing the nuclei was removed by centrifugation at 10,000 *g* at 4°C for 10 min. A bicinchoninic acid (BCA) assay was then used to determine the protein concentration of the homogenate in order that the sample contained a known concentration of 2 *μ*g of protein/*μ*L.

The sample was made up from protein, homogenizing buffer, and Laemmli SDS Buffer (3.78 g [30%] glycerol, 2.6 mL 0.625 M Tris buffer, 3 mL 20% sodium dodecyl sulfate, 0.5 mL 0.5% bromophenol blue, 0.12 mL deionized water, 100 *μ*L of *β*‐mercaptoethanol per 900 *μ*L of sample buffer). Finally, the sample was heated to 95°C for 4 min. Proteins were then loaded (45 *μ*g) and separated in a Precise Tris HEPES Gel (Thermo Scientific, Northumberland, UK) applying an 80 V constant voltage for 20 min before 40 min at 140 V. Proteins were then transferred for approximately 2 h at 25 V to a nitrocellulose membrane. Successful transfer was confirmed using Ponceau S staining (Sigma Aldrich) for 5 min before washing for 1 min in 0.1 M sodium hydroxide to destain. The membrane was blocked in 5% nonfat dried milk (NFDM) in PBST (New England Biolabs, Hertfordshire, UK) for 1 h at room temperature and following three 5 min washes in PBST (PBS in 1% tween), the membrane was then incubated overnight at 4°C in anti‐SNAP23 in 5% NFDM in PBST. Following three 5 min washes in PBST, membranes were then incubated for 1 h in 5% NFDM in PBST followed by a 1 h incubation in an appropriately targeted horseradish peroxidase–linked secondary antibody in 5% NFDM in PBST. The membrane was then washed twice in PBST (each for 5 min) followed by one 15 min wash in Tris‐buffered saline. Antibody binding was detected using enhanced chemiluminescence HRP detection reagent (GE Healthcare, Little Chalfont, Bucks, UK) and imaging was performed using Chemi Doc software (Bio‐Rad, Hemel Hempsted, Herts, UK).

### Statistics

Total mitochondria and total IMTG content and SNAP23 intensity of type I and nontype I fibers were compared using a paired samples t‐test. Colocalization of SNAP23 with IMTG, mitochondria, and the plasma membrane was investigated using both Pearson's correlation coefficient and Manders' colocalization coefficient. Statistical significance was set at *P* < 0.05. All data are expressed as mean ± SEM.

## Results

Immunofluorescence staining of SNAP23 in human skeletal muscle obtained from six lean individuals showed intense puncta stained within the cells (Fig. 2A). Some parts of the cell border regions were also stained with a greater intensity than others (Fig. 2A). When dual labeled with anti‐MHCI to denote type I fibers (Fig. 2B), there were no significant differences in SNAP23 staining intensity between type I and nontype I fibers (Fig. 2C) (*P* = 0.422).

**Figure 3:**
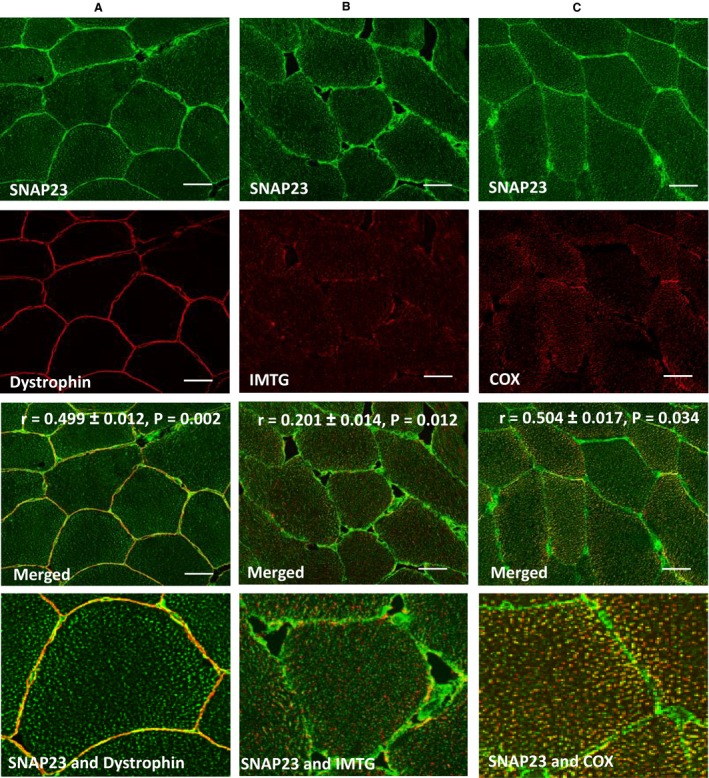
Representative images showing SNAP23 colocalization with the plasma membrane, mitochondria, and IMTG. Representative images showing SNAP23 colocalization with the plasma membrane marker dystrophin (A), mitochondrial marker COX (B), and IMTG staining using oil red O (C). All images were obtained using a 63× oil objective of a confocal microscope. Bar = 30 *μ*m. Pearson's correlation coefficients are shown on the merged images (mean ± SEM). The Pearson's correlation coefficients of the nonmatched pairs of images were; SNAP23 and Dystrophin (*r* = 0.007 ± 0.003); SNAP23 and IMTG (*r* = 0.000 ± 0.003); SNAP23 and COX (*r* = 0.006 ± 0.005). All these values were significantly lower (*P* < 0.05) than the matched image pairs demonstrating that the colocalization detected was not due to chance. The bottom panels of images highlight specific regions of interest which best visualize the overlapping yellow pixel clusters (i.e., colocalization) in plasma membrane, LDs, and mitochondria.

Immunofluorescence staining of SNAP23 in cross sections of human skeletal muscle revealed an intense stain in the 1 *μ*m layer containing the plasma membrane. Combined staining of SNAP23 with the plasma membrane marker dystrophin revealed partial colocalization with dystrophin (Pearson's *r* = 0.50 ± 0.01, *P* = 0.002) (Fig. 3A). Analyses of these images using Manders' colocalization coefficient revealed that 22 ± 2% of the pixels with a positive SNAP23 stain also stained positively for dystrophin. SNAP23 distribution at the plasma membrane was heterogeneous with some regions of the plasma membrane showing a higher degree of colocalization (higher density of yellow pixels) than others. This is most clearly visible on the larger magnification images (Fig. 3A).

Immunofluorescence staining of SNAP23 in cross sections of human skeletal muscle showed some intense punctate staining. To identify which punctate organelles contained such a high SNAP23 content the staining of SNAP23 was also combined with the neutral lipid dye oil red O and with anti‐COX as a marker of mitochondria. Quantitative analysis of the oil red O stained LDs revealed no difference in LD size between the type I (0.51 ± 0.03 *μ*m^2^) and nontype I (0.60 ± 0.07 *μ*m^2^) fibers (*P* = 0.086), whereas type I fiber LD area fraction (0.023 ± 0.002) was significantly greater than type II fiber LD area fraction (0.016 ± 0.002) (*P* = 0.004). In addition, the staining intensity of COX was greater in type I fibers (mean = 147 ± 7) compared to nontype I fibers (mean = 136 ± 7) (*P* = 0.035). Costaining of SNAP23 with oil red O showed a limited colocalization with the LDs (Pearson's *r* = 0.20 ± 0.01, *P* = 0.012) (Fig. 3B). Analysis of these images using the Manders' colocalization coefficient showed that 12 ± 2% of the pixels with a positive SNAP23 stain also stained positively with oil red O. The larger magnification image (Fig. 3B) shows that this colocalization primarily occurred in regions in close proximity to the plasma membrane at the periphery of the cell. This observation is likely to be related to previous research from our group (Shaw et al. (2008) using confocal immunofluorescence) and others (Tarnopolsky et al. (2007) using electron microscopy) showing that the density of LDs is decreasing going from the area immediately below the plasma membrane to the core of the muscle fibers. When SNAP23 was stained in combination with the mitochondrial marker COX, it became clear that the punctate SNAP23 staining colocalized to a greater extent with the mitochondria (Pearson's *r* = 0.50 ± 0.02, *P* = 0.034) (Fig. 3C) than with the LDs (Fig. 3B). The larger magnification image (Fig. 3C) showed that the colocalization of SNAP23 with COX occurred to a greater extent toward the periphery of the cell than in the core of the muscle fibers. This observation again is likely to be related to previous research from our group (Shaw et al. 2008 using confocal immunofluorescence) and others (Howald et al. 1985; Tarnopolsky et al. 2007 using electron microscopy) showing that the density of mitochondria decreases going from the area immediately below the plasma membrane to the core of the muscle fibers. Analysis of these images using the Manders' correlation coefficient revealed that 35 ± 1% of the pixels with a positive SNAP23 stain also stained positively with COX.

Furthermore, when we consider the amount of the mitochondria, LDs, and plasma membrane stains colocalized with SNAP23, it was found using Manders' correlation coefficient that 64 ± 12% of the plasma membrane was associated with SNAP23 which describes the heterogeneous distribution that we see in these regions (Fig. 3A). Furthermore, 19 ± 1% of the total LD pool colocalized with SNAP23, but a larger proportion, 31 ± 2% of the mitochondrial network is colocalized with SNAP23 in skeletal muscle of lean men (Fig. 3B and C).

## Discussion

The aim of this study was to generate detailed information on the localization of SNAP23 in human skeletal muscle. In particular, the colocalization of SNAP23 with the plasma membrane, mitochondria, and LDs was investigated.

As SNAP23 is a t‐SNARE and has been proposed to play a role in the docking and fusion of GLUT4 vesicles with the plasma membrane (Foster et al. 1999; Kawanishi et al. 2000), a high degree of colocalization with the plasma membrane was expected. This indeed was confirmed in the images (Fig. 3), which showed substantial colocalization with the plasma membrane marker dystrophin. Our data are in line with results recently obtained in human skeletal muscle from lean individuals using widefield immunofluorescence microscopy (Bostrom et al. 2010). A high SNAP23 content has also been observed in the plasma membrane of cardiomyocytes (Bostrom et al. 2007) and in plasma membrane fractions of rat adipocytes (St‐Denis et al. 1999). The larger magnification image (Fig. 3A) showed a heterogeneous nature of the colocalization of SNAP23 with the plasma membrane. A heterogeneous distribution with SNAP23 clusters being concentrated in noncaveolar, cholesterol‐rich lipid rafts has previously been observed in 3T3 L1 adipocytes (Chamberlain and Gould 2002). Future research is required to show that the sections with the high SNAP23 content (Fig. 3A) also are lipid rafts and the sites of GLUT4 fusion and docking in human skeletal muscle fibers.

As SNAP23 is a member of the SNARE protein family, it has also been proposed to be involved in other fusion processes within skeletal muscle. In this study, SNAP23 was localized in intense punctate staining (Fig. 2). As SNAP23 has been proposed to also play a role in LD fusion in fibroblasts (Bostrom et al. 2007), we also investigated SNAP23 colocalization with LDs. SNAP23 only showed a weak partial colocalization with LDs (Fig. 3B). It cannot be excluded though that colocalization of SNAP23 with LDs is more prevalent in insulin‐resistant individuals in line with the hypothesis that LDs hijack SNAP23 in obese individuals (Sollner 2007) and patients with type 2 diabetes (Bostrom et al. 2010) thereby reducing plasma membrane SNAP23 and contributing to the development of skeletal muscle insulin resistance.

The hypothesis that SNAP23 is involved in the formation of LD‐mitochondria complexes is based on recent observations in fibroblasts (Jagerstrom et al. 2009). It has been proposed that these complexes play a functional role in the channeling of FA liberated by lipolysis of LDs into neighboring mitochondria for subsequent *β*‐oxidation. The colocalization between SNAP23 and the mitochondrial stain COX observed in this study has a higher PCC than the colocalization with LDs. Furthermore, the images with a larger magnification (Fig. 3B and C) clearly show a higher density of yellow pixels and therefore a higher percentage colocalization with pixels in the mitochondrial mask than with pixels in the LD masks. This is the first evidence that SNAP23 is in close spatial contact with the mitochondria in human skeletal muscle in vivo (Fig. 3C) and might play a similar metabolic role in human skeletal muscle in vivo.

In conclusion, this study describes a detailed validation of a SNAP23 antibody for use in confocal immunofluorescence microscopy of human skeletal muscle and reveals that in the muscle of lean healthy trained men, SNAP23 primarily has a high colocalization with the plasma membrane and with the mitochondria. A weaker colocalization was observed with the LDs. The latter was stronger at the periphery of muscle fibers, where the LD content is known to be the highest (Tarnopolsky et al. 2007; Shaw et al. 2008). The observed distribution data are in line with the established traditional metabolic role of SNAP23 in GLUT4 docking with the plasma membrane and recent evidence from in vitro studies that suggest a role of SNAP23 in the shuttling of FAs generated by lipolysis of IMTG of LDs into the mitochondria for subsequent *β*‐oxidation. Further studies should investigate whether there are differences in the subcellular distribution of SNAP23 between target membranes in the muscle of trained versus sedentary individuals, obese versus lean individuals, and insulin‐resistant patients versus those with a normal glucose tolerance. Furthermore, the techniques described herein will allow exploration of the role that SNAP23 plays in the mechanisms leading to impairments in lipid metabolism and skeletal muscle insulin resistance in sedentary, obese, and aging individuals. Future research should also investigate whether SNAP23 is hijacked from the plasma membrane in obese individuals and patients with type 2 diabetes.

## Conflicts of Interest

There are no conflicts of interest.
